# Ethyl Acetate Fraction of* Amomum xanthioides* Exerts Antihepatofibrotic Actions via the Regulation of Fibrogenic Cytokines in a Dimethylnitrosamine-Induced Rat Model

**DOI:** 10.1155/2016/6014380

**Published:** 2016-08-10

**Authors:** Sung-Bae Lee, Hyeong-Geug Kim, Hyo-Seon Kim, Jin-Seok Lee, Hwi-Jin Im, Won-Yong Kim, Chang-Gue Son

**Affiliations:** Liver and Immunology Research Center, Daejeon Oriental Hospital of Oriental Medical College of Daejeon University, 176-9 Daeheung-ro, Jung-gu, Daejeon 301-724, Republic of Korea

## Abstract

*Amomum xanthioides* has been traditionally used to treat diverse digestive system disorders in the Asian countries. We investigated antihepatofibrotic effects of ethyl acetate fraction of* Amomum xanthioides* (EFAX). Liver fibrosis is induced by dimethylnitrosamine (DMN) injection (intraperitoneally, 10 mg/kg of DMN for 4 weeks to Sprague-Dawley rats). EFAX (25 or 50 mg/kg), silymarin (50 mg/kg), or distilled water was orally administered every day. The DMN injection drastically altered body and organ mass, serum biochemistry, and platelet count, while EFAX treatment significantly attenuated this alteration. Severe liver fibrosis is determined by trichrome staining and measurement of hydroxyproline contents. EFAX treatment significantly attenuated these symptoms as well as the increase in oxidative by-products of lipid and protein metabolism in liver tissues. DMN induced a dramatic activation of hepatic stellate cells and increases in the levels of protein and gene expression of transforming growth factor-beta (TGF-*β*), platelet derived growth factor-beta (PDGF-*β*), and connective tissue growth factor (CTGF). Immunohistochemical analyses revealed increases in the levels of protein and gene expression of *α*-smooth muscle actin. These alterations were significantly normalized by EFAX treatment. Our findings demonstrate the potent antihepatofibrotic properties of EFAX via modulation of fibrogenic cytokines, especially TGF-*β* in the liver fibrosis rat model.

## 1. Introduction

Liver fibrosis is a pathological consequence of the wound healing response to chronic liver injuries, leading to the excessive accumulation of extracellular matrix (ECM) protein in hepatic tissues [1]. Liver fibrosis can be induced by various causes, including chronic alcohol abuse, hepatitis viral infections, metabolic disorders, and autoimmune disease [2, 3]. Liver fibrosis is reversible in certain conditions but can commonly progress to liver cirrhosis, the final step in liver fibrosis, if no proper treatment is given [4]. Worldwide, 2.2% of total deaths were caused by liver cirrhosis in 2013 [5].

Therefore, the development of liver fibrosis is critical with respect to the clinical outcome of patients with chronic liver injuries. ECMs such as *α*-smooth muscle actin (*α*-SMA) and collagens are generated by activated hepatic stellate cells (HSCs) [6]. “Quiescent” HSCs convert to myofibroblasts via activation by fibrogenic cytokines, including transforming growth factor- (TGF-) *β*, platelet derived growth factor- (PDGF-) *β*, and connective tissue growth factor (CTGF) [7]. Accordingly, the inhibition of the HSC activation or the modulation of the above three fibrogenic cytokines is therapeutic target for treatment of liver fibrosis.

Meanwhile,* Amomum xanthioides* Wall. ex Baker (Amomi Fructus) is a well-known medicinal herb that has been used clinically to treat digestive system disorders for more than a thousand years in Asia.* A. xanthioides* has been traditionally used to treat indigestion, diarrhea, and flatulence in China [8], Japan [9], and Thailand [10], which are the common complaints in patients with chronic liver diseases. We previously reported the hepatoprotective effect of* A. xanthioides* in a thioacetamide and a bile duct-ligation model, as well as the anti-inflammatory effects in a gastritis model [11–13]. In addition,* A. xanthioides* has been widely prescribed for the treatment of various liver diseases [14, 15]. Further studies however have been required, especially regarding the practical dose and a detailed explanation of the pharmacological actions of* A. xanthioides*. We therefore compared the antihepatofibrotic capacities of several* A. xanthioides* fractions based on* in vitro* experiments and determined the ethyl acetate fraction of* Amomum xanthioides* (EFAX) with the most potent pharmacological activity at relatively very low concentrations.

We herein investigated the antihepatofibrotic effects of a low-dose EFAX and explored the underlying mechanisms in rat model of DMN-induced liver fibrosis.

## 2. Materials and Methods

### 2.1. Reagents and Chemicals

Dimethylnitrosamine (DMN), hydroxyproline,* p*-dimethylaminobenzaldehyde, 1,1,3,3-tetraethoxypropane (TEP), chloramine-T, potassium chloride (KCl), Folin-Ciocalteu's phenol reagent, and hydrochloric acid (HCl) were purchased from Sigma (St. Louis, MO). Thiobarbituric acid (TBA) was purchased from Lancaster Co. (Lancashire, UK). Histofine was purchased from Nichirei Biosciences (Tokyo, Japan). Sodium carbonate was purchased from Kanto perchloric acid and aluminum chloride was purchased from Junsei Chemical (Tokyo, Japan).

### 2.2. Preparation of Fractions for* A. xanthioides*

Korean Pharmacopoeia standard Amomi Fructus (a dried fruit of* A*.* xanthioides*) was purchased from Jeong-Seong Pharmacy (Daejeon, South Korea), and its identity was confirmed by professor Sang-Hoon Oh (Daejeon University). The* Amomum xanthioides* were washed twice using tap water and rinsed with distilled water (DW). The sample was then completely dehydrated by drying in an oven overnight (60°C). After drying, 10 kg samples of* A. xanthioides* were boiled in 100 L of DW for 3 h at 100°C, centrifuged (3,000 ×g) for 20 min, and then filtered. We firstly obtained the water extract of* Amomum xanthioides* (WAX) and the final yield (w/w) was 1.12% (total 112 g, voucher specimen number WAX-2014-W007).

**Figure 1:**
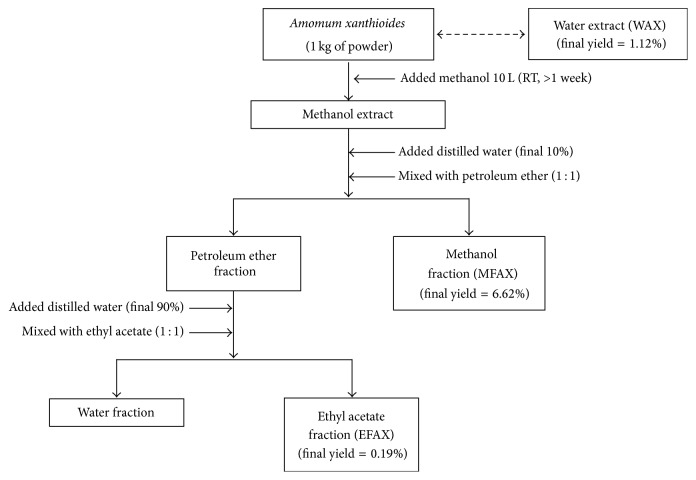
Scheme for preparation of EFAX.

To obtain the methanol and ethyl acetate fractions of* A. xanthioides*, we used an organic solvent extraction method (Figure 1). Briefly, 10 kg of* A. xanthioides* was ground and extracted in 100 L of absolute methanol for 7 days with shaking. On the 7th day, 100 mL DW was added to 900 mL methanol extract. Next, the extracts were further fractionated three times with petroleum ether (3 × 1 L) to isolate the methanol fraction of* Amomum xanthioides* (MFAX). Then, 100 mL of the petroleum ether extract was mixed with 900 mL DW (3 × 1 L) and further fractionated two times with ethyl acetate (2 × 1 L) to isolate the ethyl acetate fraction of* Amomum xanthioides* (EFAX). Finally, we obtained a portion of the 100% MFAX and EFAX. The final fraction yields were 6.62% (w/w) for MFAX (total 662 g, voucher specimen number MFAX-2014-MF001) and 0.19% (w/w) for EFAX (total 19 g, voucher specimen number EFAX-2014-EF002). WAX, MFAX, and EFAX were stored at −70°C and dissolved in DW for the experiments.

### 2.3. Fingerprinting Analysis of WAX, MFAX, and EFAX

**Figure 2:**
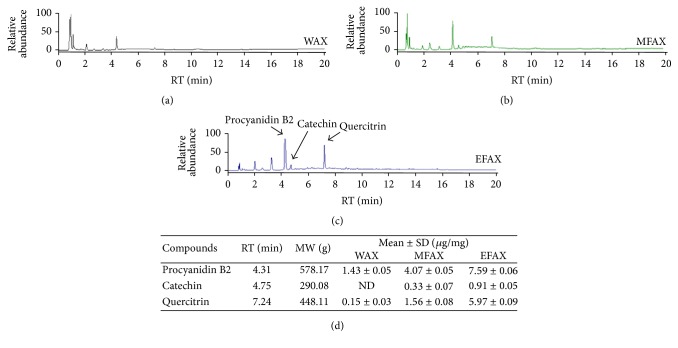
Fingerprinting analysis. WAX, MFAX, and EFAX were subjected to UHPLC-MS/MS. Chromatogram of (a) WAX, (b) MFAX, and (c) EFAX. (d) Quantitative analysis of the WAX, MFAX, and EFAX.

To determine the reproducibility of WAX, MFAX, and EFAX samples, fingerprinting was performed using ultra-high-performance liquid chromatography-tandem mass spectrometry (UHPLC-MS/MS). Five milligram aliquots of the WAX, MFAX, and EFAX samples were dissolved in 1 mL 90% methanol, and the solution was filtered. Sample solutions of 10 *μ*L were subjected to UHPLC-MS/MS using an LTQ Orbitrap XL linear ion-trap MS Spectrometer (San Jose, CA). Separation was performed on an Accela UHPLC system using an Acquity BEH C18 column (1.7 *μ*m, 100 × 2.1 mm; Waters, Milford, Connecticut). The column was eluted at a flow rate of 0.4 mL/min using water (in 0.1% formic acid) and acetonitrile (in 0.1% formic acid), which were used as mobile phases A and B, respectively. The following gradients were applied: 0-1 min, 0-1% B in A; 1–7 min, 1–100% B in A; 7–10 min, 100–1% B in A (linear gradient). The compositional analyses were conducted using a photodiode array at 200–600 nm. The full-scan mass spectra were acquired at 150–1500 *m*/*z* in positive and negative modes. An Orbit rap analyzer was used for high-resolution mass data acquisition with a mass resolving power of 30,000 FWHM at 400 *m*/*z*. Tandem mass (MS/MS) spectra were acquired in data-dependent mode by collision-induced dissociation. The quantitative analysis of the major three compounds in EFAX, including procyanidin B2, catechin, and quercitrin, was performed using UHPLC-MS/MS (Figure 2).

### 2.4. Determination of Total Flavonoid and Phenolic Contents

The total flavonoid contents of WAX, MFAX, and EFAX were measured using a previously developed method [16]. Briefly, 0.5 mL solutions of each sample (10% w/v in absolute methanol) were separately mixed in a flavonoid assay buffer (1.5 mL methanol, 100 *μ*L 10% aluminum chloride, 100 *μ*L 1 M potassium acetate, and 2.8 mL DW). After 30 min of incubation at room temperature, 200 *μ*L of the mixture was transferred to a 96-well plate, and absorbance was measured at 415 nm using a spectrophotometer (Palo Alto, CA). The calibration curve was obtained by preparing quercetin solutions at concentrations from 12.5 to 100 *μ*g/mL in methanol. The total flavonoid contents values were expressed in terms of quercetin equivalent (mg/g of dry mass), which is a common reference for flavonoid.

The total phenolic contents were measured using the Folin Ciocalteu method [17]. The WAX, MFAX, and EFAX samples were mixed with 2.5 mL 0.2 N Folin-Ciocalteu's phenol reagent for 5 min, after which 2 mL 75 g/L sodium carbonate was added. After 2 h of incubation at room temperature, the optical density of the reaction product was read at 760 nm using a spectrophotometer. The standard curve was prepared using 50 to 250 mg/mL solutions of gallic acid in a methanol-water solution (1 : 1, v/v). The total phenolic contents values were expressed in terms of gallic acid equivalent (mg/g of dry mass), which is a common reference for phenolic contents.

### 2.5. Animals and Experimental Design

A total of 30 male Sprague-Dawley rats (6 weeks old, 160–180 g) were purchased from Daehanbiolink (Choong-book, South Korea). Seven days of acclimation was allowed at 22 ± 2°C under a 12 h light/12 h dark cycle. All of the animals had free access to water and standard chow diet. After acclimation, all of the rats were divided into five groups (*n* = 6 for each group) and orally administered with DW, EFAX (25 or 50 mg/kg), or silymarin (50 mg/kg) daily for 4 weeks. To induce liver fibrosis, 10 mg/kg DMN was intraperitoneally injected on 3 consecutive days per week for 4 weeks. The groups were as follows: (1) naive group (DW with 0.9% neutral saline), (2) control group (DW with 10 mg/kg DMN), (3) EFAX 25 (25 mg/kg EFAX with 10 mg/kg DMN), (4) EFAX 50 (50 mg/kg EFAX with 10 mg/kg DMN), and (5) silymarin 50 (50 mg/kg silymarin with 10 mg/kg DMN). The naive group was also intraperitoneally injected with same volume of 0.9% neutral saline for 4 weeks. Body weight was measured twice a week and once shortly before sacrifice.

On the final day of the experiment, the animals were sacrificed under ether anesthesia, and whole blood was isolated from the abdominal aorta. The liver and spleen tissues were removed and weighed and then collected for biochemical analyses and other measurements. The animal experiment was conducted in accordance with the Guide for the Care and Use of Laboratory Animals prepared by the US National Institutes of Health and was approved by the Institutional Animal Care and Use Committee of Daejeon University (DJUARB2015-007).

### 2.6. Serum Biochemical Analysis

Whole blood was isolated from the abdominal aorta and transferred to an EDTA-coated tube (Plymouth, UK). Serum samples were obtained for subsequent separation after 1 hour of blood clotting using Vacutainer tubes (Plymouth, UK). The platelet counts in each sample were measured using a HEMA VET 850 automatic analyzer (Oxford, CT). The serum was separated by centrifugation (3,000 ×g, 15 min) following blood clotting. The serum levels of aspartate transaminase (AST), alanine transaminase (ALT), and total bilirubin were determined using an Auto Chemistry Analyzer (Emeryville, CA).

### 2.7. Histomorphology and Immunohistochemistry for *α*-Smooth Muscle Actin (*α*-SMA)

On the final day of the experiment, the liver tissues were removed and weighed. The tissue samples were fixed with a 10% neutral formalin solution. The tissues then underwent general processing. Paraffin-embedded liver tissues were sectioned (5 *μ*m) and stained with hematoxylin and eosin (H&E) or Masson's trichrome dye for histopathological evaluation. Immunohistochemistry was performed with an anti-*α*-SMA mouse monoclonal antibody (Cambridge, UK) and a Vectastain ABC kit (Vector, Burlingame, CA). The samples were visualized with Tetramethylbenzidine (TMB) substrate and examined under an optical microscope (×100 magnification).

The liver histological examination was examined and graded by two independent investigators who were blind to samples' groups. The samples were graded according to published criteria for magnitude analysis and inflammation. The histomorphological changes for inflammation were also scored (×100) using H&E staining (grade 0: naive, absence of pathology to <5% of maximum pathology; grade 1; <10% of maximum pathology; grade 2; 15% to 20% of maximum pathology; grade 3, >20% of maximum pathology) [18]. A METAVIR fibrosis score from 0 to 4 was used to differentiate the levels of liver fibrosis. Briefly, stage 0 indicates no scarring, stage 1 indicates minimal scarring, stage 2 indicates scarring that extends outside the vascularized area of the liver, stage 3 indicates bridging fibrosis that has spread and connected to fibrotic areas, and stage 4 indicates advanced scarring of the liver or cirrhosis [19]. The number of *α*-SMA positive cells (stained blue-violet color) was also calculated and expressed as a fold change after normalization to the naive group. The inflammation and METAVIR scores and the number of *α*-SMA positive cells were calculated using ImageJ analysis software v. 1.67 (NIH, Rockville, MD).

### 2.8. Determination of Hydroxyproline in Liver Tissues

Hydroxyproline determination was performed with a slight modification of a previously method described [20]. Briefly, liver tissues (200 mg) stored at −70°C were homogenized in 2 mL 6 N HCl and incubated overnight at 110°C. After filtering the acid hydrolysates using a 0.45 *μ*m filter (Tokyo, Japan), 50 *μ*L samples or hydroxyproline standards in 6 N HCl were incubated at 60°C to dry. The dried samples were dissolved with methanol (50 *μ*L), after which 1.2 mL 50% isopropanol and 200 *μ*L of chloramine-T solution were added to each same sample. The samples were then incubated at room temperature for 10 min. After incubation,* Ehrlich's* solution (1.3 mL) was added, and the samples were further incubated at 50°C for 90 min. The optical density of the reaction product was read at 558 nm using a spectrophotometer. A standard curve was constructed using serial twofold dilutions of a 1 mg hydroxyproline solution.

### 2.9. Determination of Lipid Peroxidation and Protein Carbonyl Contents in Liver Tissues

The levels of malondialdehyde (MDA; the final product for lipid peroxidation) in the liver tissues were determined using TBA reactive substances method (TBARS), as described previously [21]. The concentration of TBARS was expressed as *μ*mol per gram of tissue, using TEP as a standard. The protein carbonyl contents in the liver tissues were determined according to the manufacturer's protocol [22].

### 2.10. Determination of the Levels of TGF-*β*1, PDGF-BB, CTGF, and TIMP-1 in Liver Tissues

One hundred milligrams of liver tissues was homogenized with RIPA buffer and centrifuged at 10,000 ×g for 15 min at 4°C. The supernatant fraction was used to determine the levels of fibrosis-related cytokines. The protein levels of TGF-*β*1, PDGF-BB, and tissue inhibitor of matrix metalloprotease (TIMP) in the liver tissue were also determined using an ELISA kit (R&D Systems, Minneapolis, MN). The quantification of CTGF was performed using a modification of the sandwich ELISA method described previously [23]. Briefly, a 96-well ELISA plate was coated with 100 mL of goat polyclonal anti-rat antibody at a concentration of 10 mg/mL in PBS and 0.02% sodium azide overnight. After incubation with blocking buffer (PBS, 0.02% sodium azide and 1% bovine serum albumin) and washing (four times), 50 mL of the sample or recombinant human CTGF standard was added for 1 hour. Then, 100 mL of the primary rabbit polyclonal anti-goat antibody (2 mg/mL) and 50 mL of the secondary donkey anti-rabbit IgG-HRP antibody (both 1 : 2000) were added (Santa Cruz Biotechnology, Germany). CTGF was quantified by measuring the absorbance at 405 nm after mixing the substrate solution and stopping solution (2 N H_2_SO_4_).

### 2.11. Real-Time PCR for Gene Expression in HSC-T6 Cells or Liver Tissues

HSC-T6 cells (2 × 10^6^) were seeded in six-well plates with 2 mL DMEM with 10% FBS and incubated overnight at 37°C and 5% CO_2_. The cell culture media was then changed to serum-free DMEM. Next, each sample of WAX, MFAX, or EFAX (25 or 50 *μ*g/mL dissolved in methanol initially and then DMEM) was added to the wells. After 6 h of incubation with the samples, 1 ng/mL TGF-*β*1 was treated for 12 hours. The mRNA of HSC-T6 cells or liver tissues was isolated using QIAzol reagent (Germantown, MD) and used for complementary DNA (cDNA) synthesis using standard protocols. Real-time PCR was performed using an iQ5 instrument. The primer sequences were used that is given in Table 1. *β*-actin levels were used for normalization.

### 2.12. Western Blot Analysis

HSC-T6 cells (2 × 10^6^) were seeded in six-well plates with 2 mL DMEM with 10% FBS and incubated overnight at 37°C and 5% CO_2_. The cell culture media was then changed to serum-free DMEM. Next, each sample of WAX, MFAX, or EFAX (25 or 50 *μ*g/mL dissolved in methanol initially and then DMEM) was added to the wells. After 6 h of incubation with the samples, 1 ng/mL TGF-*β*1 was treated for 24 hours. The protein of HSC-T6 was extracted by RIPA buffer. Each sample was separated by 10% polyacrylamide gel electrophoresis and transferred to polyvinylidene fluoride membranes. After blocking in 5% skim milk, the membranes were probed overnight at 4°C with primary antibodies (collagen type 1, *α*-SMA, and *β*-actin).

### 2.13. Statistical Analysis

The results are expressed as the means ± SD (standard deviation, *n* = 6). Statistical significance was analyzed by one-way analysis of variance (ANOVA) followed by the Tukey HSD (honest significant difference) post hoc test. In all of the analyses, *p* < 0.05, *p* < 0.01, or *p* < 0.001 was taken to indicate statistical significance.

## 3. Results

### 3.1. Compositional Analysis of WAX, MFAX, and EFAX

The compositional analysis for the main chemicals components of WAX, MFAX, and EFAX was performed using UHPLC-MS/MS. The histogram indicated that three types of flavonoid family chemicals were detected, including procyanidin B2, catechin, and quercitrin at 4.31, 4.75, and 7.24 min of retention time, respectively. The molecular weights of above chemicals were confirmed using UHPLC-MS/MS analysis and were as follows: procyanidin B2 290.08, catechin 578.17, and quercitrin 448.11. From the quantitative analysis, EFAX contained the highest amount of the above three chemical components than the other extracts (Figure 2).

### 3.2. Comparisons of the WAX, MFAX, and EFAX in Total Flavonoid and Phenolic Contents

**Figure 3:**
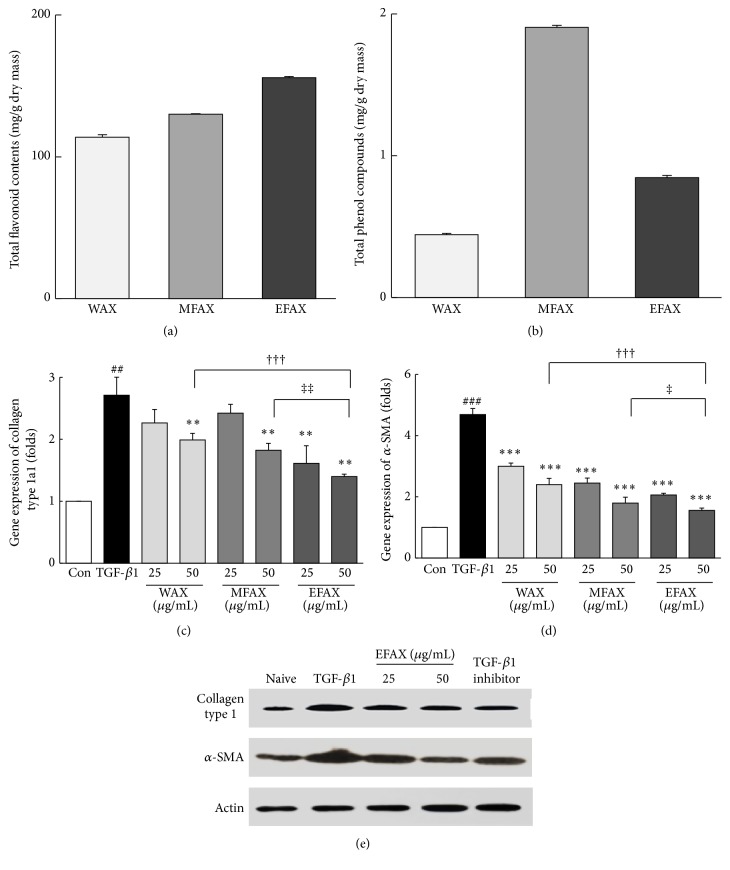
Total flavonoid and phenolic contents and mRNA expression and protein levels in HSC-T6. (a) Total flavonoid contents values are expressed in terms of quercetin equivalent (mg/g of dry mass) and (b) the total phenolic contents values are expressed in terms of gallic acid equivalent (mg/g of dry mass). The analysis of mRNA expression levels was performed for (c) Col 1a1 and (d) *α*-SMA using real-time PCR in HSC-T6 cells. (e) The protein levels of Col 1 and *α*-SMA are examined using western blot. The data are expressed as the mean ± SD (*n* = 4). ^##^ *p* < 0.01, ^###^ *p* < 0.001 compared to the control group; ^*∗∗*^ *p* < 0.01, ^*∗∗∗*^ *p* < 0.001 compared to the TGF-*β*1 treatment group; ^†††^ *p* < 0.001 comparison of WAX 50 and EFAX 50; ^†^ *p* < 0.05, ^‡‡^ *p* < 0.01 comparison of MFAX 50 and EFAX 50.

EFAX contained the most flavonoid contents (151.7 ± 0.8 mg/g) compared to WAX (113.9 ± 0.4 mg/g) and MFAX (130.1 ± 1.9 mg/g). In contrast to the total flavonoid contents, the total phenolic contents were the highest in MFAX (1.8 ± 0.0 mg/g) compared to WAX (0.8 ± 0.0 mg/g) and EFAX (1.7 ± 0.1 mg/g, Figures 3(a) and 3(b)).

### 3.3. Comparisons of WAX, MFAX, and EFAX on Antifibrotic Gene Expressions and Proteins

As measures of antihepatofibrotic properties of WAX, MFAX, and EFAX, the expression levels of two fibrosis-related genes, collagen type 1a1 and *α*-SMA, were examined following treatment in rat derived HSC-T6 cells. Treatment with TGF-*β*1 led to a remarkable upregulation of these genes (approximately 2.9-and 4.7-fold for collagen type 1a1 and *α*-SMA, resp.). Pretreatment with WAX, MFAX, or EFAX significantly reduced the expression levels of collagen type 1a1 (*p* < 0.01 for WAX 50, MFAX 50, all EFAX treatments) and *α*-SMA (*p* < 0.001 for all pretreatments) compared with the TGF-*β*1 treatment group. The effects of extracts were strongest in the EFAX 50 pretreatment group compared to WAX 50 (*p* < 0.001 in all of parameter) or MFAX 50 (*p* < 0.01 and 0.05 in collagen type 1a1 and *α*-SMA) treatment groups (Figures 3(c) and 3(d)). Treatment with TGF-*β*1 considerably increased the protein levels of collagen type 1 and a-SMA in HSC-T6 cells. Pretreatment with EFAX (especially EFAX 50) dramatically normalized those abnormalities (Figure 3(e)).

### 3.4. Effects of EFAX on DMN-Induced Changes in Body and Organ Weights

DMN injection drastically decreased the body weights (0.7-fold) compared with the naive group. The absolute liver weights were slightly reduced (0.9-fold), but the relative liver weights were considerably increased by DMN injection (1.2-fold), compared with naive group. The DMN injection caused remarkable increases in both the absolute and relative spleen weights (1.9- and 2.6-fold compared with naive group, resp.). Compared to DMN group, the administration of EFAX tended to ameliorate the above alterations without significances for all of parameters (Table 2). Silymarin (50 mg/kg), used as a reference drug, did not positively affect all of the parameters.

### 3.5. Effects of EFAX on Serum Biochemical Parameters and Platelet Counts

Compared with the naive group, the DMN injection dramatically increased the serum levels of AST, ALT, and total bilirubin, approximately 4.9-, 15.5-, and 12.0-fold, respectively. In contrast, the administration of EFAX significantly decreased the above abnormal elevations of serum ALT (*p* < 0.01 for only EFAX 25) and total bilirubin levels (*p* < 0.001 for EFAX 25 and 50) compared with the DMN group (but not AST). The DMN also injection drastically depleted the platelet counts 4.3-fold compared with naive group, while this effect was significantly attenuated by EFAX treatment (especially, EFAX 25) compared to DMN group (*p* < 0.001, Table 2). Silymarin did not show positive effects on liver enzyme serum levels or platelet counts.

### 3.6. Effects of EFAX on Histopathological Findings

**Figure 4:**
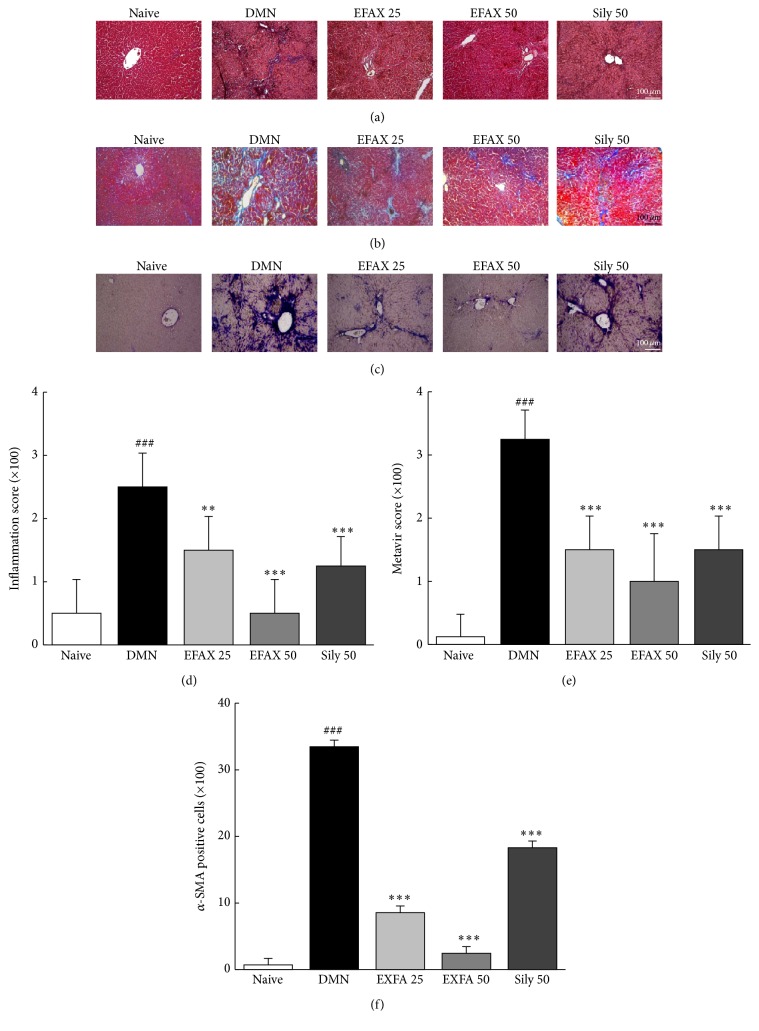
Histopathological findings and immunohistochemical staining of liver tissues. (a) Hematoxylin and eosin staining (H&E), (b) Masson's trichrome staining, and (c) immunohistochemistry for *α*-SMA; the histological examinations were performed under light microscopy (×100). (d) The inflammation scores, (e) METAVIR scores, and (f) the number of *α*-SMA positive cells were analyzed. The data are expressed as the mean ± SD (*n* = 6). ^###^ *p* < 0.001 compared with the naive group; ^*∗∗*^ *p* < 0.01, ^*∗∗∗*^ *p* < 0.001 compared with the DMN group.

DMN group showed marked bridging necrosis, inflammation, and wide infiltration of inflammatory cells around the central vein in H&E staining analysis, whereas EFAX administration drastically ameliorated these alterations (Figures 4(a) and 4(d)). Masson's trichrome staining was performed to analyze collagen synthesis in liver tissues. The results showed that hepatofibrotic changes (blue) were considerable in the DMN group, while EFAX treatment notably inhibited collagen synthesis in liver tissues (Figures 4(b) and 4(e)). To investigate HSC activation, *α*-SMA levels were analyzed by immunohistochemistry. Strong *α*-SMA signals (blue-violet) were observed in the DMN group; these signals were considerably reduced by EFAX administration (Figures 4(c) and 4(f)). The quantitative analyses of the above observations showed that EFAX treatment had statistically significant effects compared with the DMN group (*p* < 0.01 and *p* < 0.001 for EFAX 25 and EFAX 50 in inflammation score; *p* < 0.001 for all EFAX treatments in both the* Metavir'* score and *α*-SMA positive signal). Administration of silymarin also moderately attenuated these morphological alterations (*p* < 0.001).

### 3.7. Effects of EFAX on Hydroxyproline, Lipid Peroxidation, and Protein Carbonyl Contents in Liver Tissues

**Figure 5:**
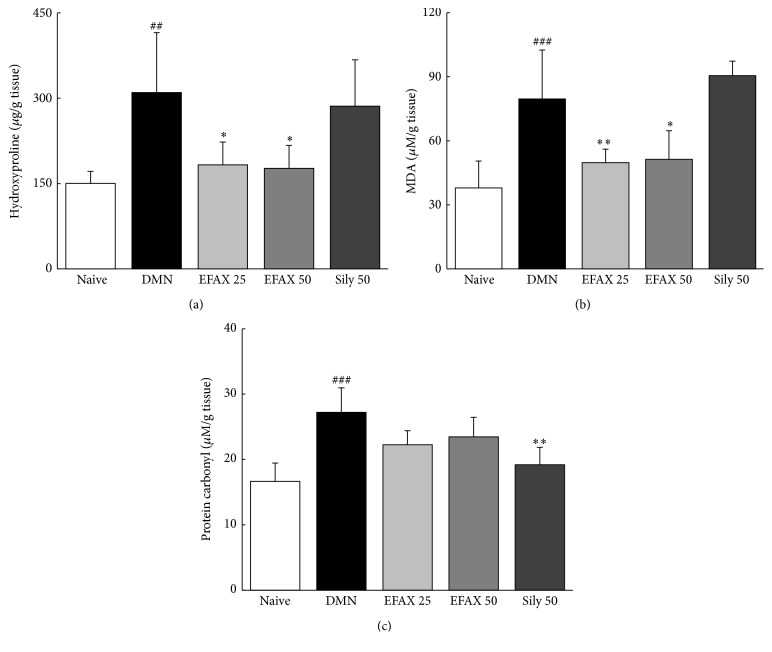
Contents of hydroxyproline, MDA, and protein carbonyl in liver tissues. (a) Hydroxyproline, (b) MDA, and (c) protein carbonyl contents were determined in the liver tissues. The data are expressed as the mean ± SD (*n* = 6). ^##^ *p* < 0.01, ^###^ *p* < 0.001 compared with the naive group; ^*∗*^ *p* < 0.05, ^*∗∗*^ *p* < 0.01 compared with the DMN group.

DMN injection markedly increased the hydroxyproline contents 2.1-fold compared with naive group, whereas this effect was significantly ameliorated by administration with EFAX (*p* < 0.05 for EFAX 25 and 50, Figure 5(a)). DMN injection also induced considerable increases in MDA (final product of lipid peroxidation) and protein carbonyl contents 2.1- and 1.7-fold, compared to naive group, whereas administration with EFAX significantly decreased abnormal elevations of MDA level (*p* < 0.01 and 0.05 for EFAX 25 and 50) and showed a decreasing tendency of protein carbonyl contents (*p* > 0.05, Figures 5(b) and 5(c)). Silymarin significantly decreased hepatic protein carbonyl contents (*p* < 0.01), but no significant effect was observed for MDA or hydroxyproline contents.

### 3.8. Effects of EFAX on Fibrogenic Cytokines and TIMP-1 Levels in Liver Tissues

**Figure 6:**
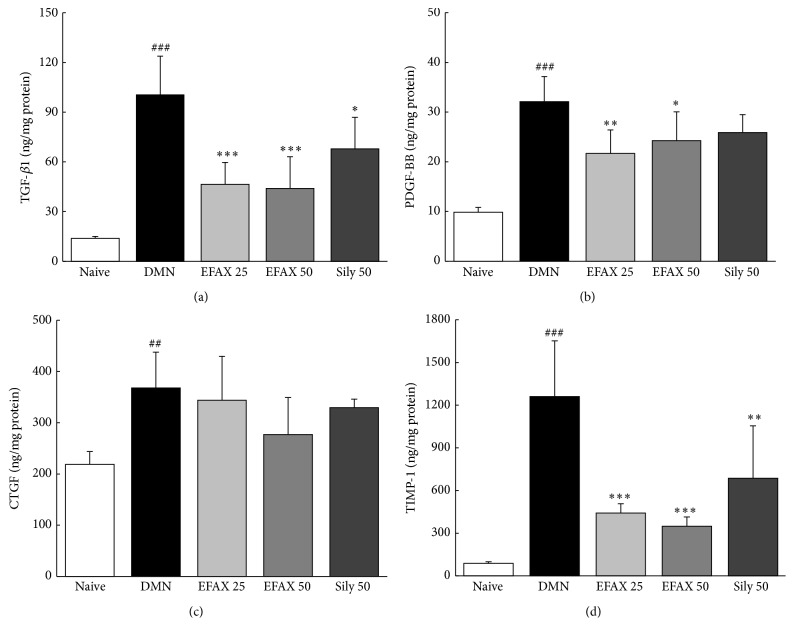
Determination of fibrogenic cytokines and TIMP-1 levels in liver tissues. The quantitative analysis of (a) TGF-*β*1, (b) PDGF-BB, (c) CTGF, and (d) TIMP-1 was performed in liver tissues using ELISA kits. The data are expressed as the mean ± SD (*n* = 6). ^##^ *p* < 0.01, ^###^ *p* < 0.001 compared with the naive group; ^*∗*^ *p* < 0.05, ^*∗∗*^ *p* < 0.01, ^*∗∗∗*^ *p* < 0.001 compared with the DMN group.

Compared with naive group, DMN injection drastically elevated the levels of profibrogenic cytokines, including TGF-*β*1, PDGF-BB, and CTGF 7.7-, 3.5-, and 1.7-fold, respectively. The administration of EFAX significantly decreased hepatic protein levels of TGF-*β*1 (*p* < 0.001 for EFAX 25 and 50) and PDGF-BB (*p* < 0.01 and 0.05 for EFAX 25 and 50) and reduced CTGF levels (*p* > 0.05) compared with the DMN group (Figures 6(a)–6(c)). The protein levels of TIMP-1 in liver tissues were increased 14.0-fold compared with the naive group, whereas this abnormal elevation was significantly ameliorated by administration with EFAX (*p* < 0.001 for EFAX 25 and 50, Figure 6(d)). Silymarin treatment resulted in significant decreases in the protein levels of TGF-*β*1 (*p* < 0.05) and TIMP-1 (*p* < 0.01) in liver tissues (but *p* > 0.05 for PDGF-BB and CTGF).

### 3.9. Effects of EFAX on Gene Expression in Liver Tissues

**Figure 7:**
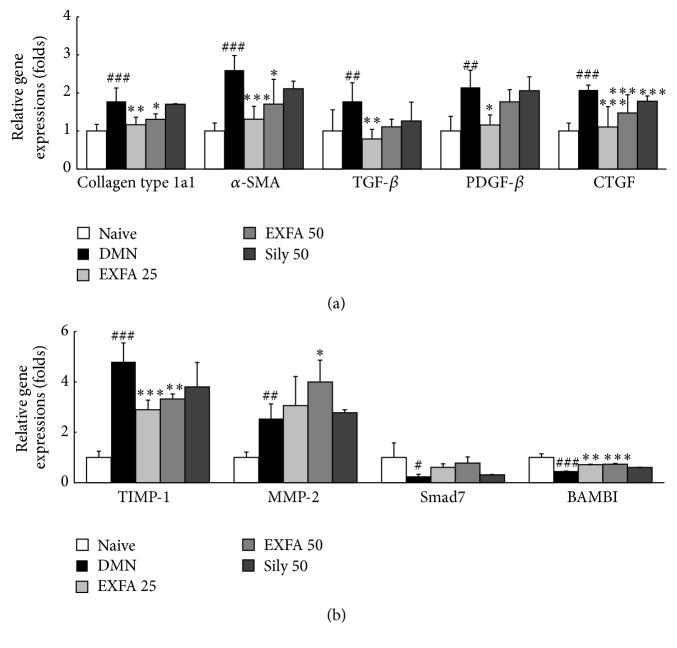
The mRNA expression levels of liver fibrosis-related genes in liver tissues. The analyses of mRNA expression levels were performed to determine the mRNA levels of (a) collagen type 1a1, *α*-SMA, TGF-*β*, PDGF-*β*, and CTGF and (b) TIMP-1, MMP-2, BAMBI, and Smad7 using real-time PCR. Gene expression is presented with the level in the naive group set as 1 after normalization to *β*-actin. The data are expressed as the mean ± SD (*n* = 6). ^##^ *p* < 0.01, ^###^ *p* < 0.001 compared to the naive group; ^*∗*^ *p* < 0.05, ^*∗∗*^ *p* < 0.01, ^*∗∗∗*^ *p* < 0.001 compared to the DMN group.

Compared with naive group, the gene expression levels of ECM, including collagen type 1a1 and *α*-SMA, in liver tissues were markedly elevated approximately 1.8- and 2.6-fold, respectively, in DMN group. DMN injection also significantly upregulated the gene expression levels of fibrogenic cytokines, including TGF-*β*, PDGF-*β*, and CTGF, 1.8-, 2.2-, and 2.1-fold, respectively. Compared with naive group, the DMN injection caused drastic upregulations of two ECM turnover-related genes, TIMP-1 and MMP-2, 4.8- and 2.5-fold. In the DMN group, the gene expression levels of TGF-*β*1 antagonists, such as Smad7 and BAMAI, were remarkably lowered 0.3- and 0.5-fold, respectively, compared with the naive group. The administration with EFAX significantly normalized gene expression levels of collagen type 1a1 (*p* < 0.01 and 0.05 for EFAX 25 and 50), *α*-SMA (*p* < 0.001 and 0.05 for EFAX 25 and 50), and fibrogenic cytokines (TGF-*β*: *p* < 0.01 for EFAX 25, PDGF-*β*: *p* < 0.05 for EFAX 25, and CTGF: *p* < 0.001 for all EFAX treatments) compared with the DMN group (Figure 7(a)). Moreover, gene expression levels of TIMP-1 (*p* < 0.001 and 0.01 for EFAX 25 and 50), MMP-2 (*p* < 0.05 for EFAX 50), and BAMBI (*p* < 0.01 and *p* < 0.001 for EFAX 25 and 50) were significantly normalized by EFAX treatment, also gene expression level of Smad7 showed tendency to normalization by EFAX treatment (Figure 7(b)). Silymarin treatment also significantly normalized the gene expression level of CTGF (*p* < 0.001), but not significant for other parameters.

## 4. Discussion

Many groups have previously attempted to develop antihepatofibrotic therapeutics. Many candidates, such as interferon-*γ*, angiotensin II antagonist, and ursodeoxycholic acid, have shown potent antihepatofibrotic effects in animal models. However, such treatments have failed to demonstrate any beneficial effects clinically because they lack antihepatofibrotic effects [24–26]. In order to support the clinical relevance of traditional use of* Amomum xanthioides* and evaluate its potential as an antihepatofibrotic drug, the present study investigated the pharmaceutical action and underlying mechanisms of EFAX, the most potent fraction of* Amomum xanthioides* extract.

We adapted a DMN-induced rat hepatofibrosis model for present study. DMN is a well-known chemotoxin that is used in experimental model of liver fibrosis [27]. As expected, DMN injection considerably elevated the serum levels of liver enzymes and total bilirubin. In addition, DMN injection caused drastic reduction body weight, splenomegaly, and thrombocytopenia, all of which are typical characteristics of liver cirrhosis [3, 28]. These results indicated the successful induction of hepatocyte destruction and inflammation as well as hepatic fibrosis, which was evidenced by the infiltration of inflamed cells in H&E staining and fibrotic changes in Masson's trichrome staining (Table 2 and Figures 4(a) and 4(b)). The severity of hepatic injury in present study was severer than our previous study (same dose and period of DMN treatment, but using Wistar rat instead of SD rat). The administration of EFAX considerably attenuated the above abnormalities in the liver enzyme levels and histological finding.

The fibrotic changes observed in our model occurred just prior to cirrhosis, as the METAVIR fibrosis score was greater than > 3. The score was decreased to less than 2 by administration with EFAX (Figures 4(b) and 4(e)). The antihepatofibrotic effect of EFAX corresponded well with the quantitative measurement of hydroxyproline contents (Figure 5(a)). EFAX treatment also attenuated the DMN-induced oxidative alterations in hepatic tissues, as evidenced by measurement of final product for lipid peroxidation (Figure 5(b)). It is well known that oxidative stress contributes to pathological changes that are characterized by hepatic fibrosis via continuous damage to hepatocytes [29, 30].

To investigate the underlying mechanisms of EFAX treatment, we examined its pharmacological activity with respect to HSCs and the primary fibrogenic cytokines. HSCs perform a central role in the development of liver fibrosis via the production of ECM in hepatic tissues [31]. Chronic liver damage alters HSCs from a quiescent state to activated state under stimulation of three primary fibrogenic cytokines, including TGF-*β*, PDGF-*β*, and CTGF. These cytokines induce the activation and proliferations of HSCs, which consequently result in accumulate excessive ECM in the liver [32]. In the present study HSC activation by DMN injection was observed immunohistochemically by staining for *α*-SMA, a potent marker of HSC activation [33]. EFAX efficiently inhibited HSC activation (Figures 4(c) and 4(f)). EFAX treatment also significantly normalized the dramatic DMN-induced increases in the levels of the two fibrogenic cytokines (TGF-*β* and PDGF-*β*), and these effects were observed at both the protein and mRNA level (Figures 6(a), 6(b), and 7(a)). TGF-*β* has the most central role in HSC activation, acting both directly and indirectly to induce the expression of PDGF-*β*, as well as CTGF receptors in hepatocytes or HSCs during liver fibrosis [34, 35]. In the development liver fibrosis, PDGF-*β* acts as a potent mitogen or activator of HSCs, and CTGF mediates TGF-*β*-induced ECM formation in liver tissues [36, 37]. These results were in accordance with proteins assays for collagen type 1 and *α*-SMA in HSC-T6 cells under TGF-*β* stimulation (Figure 3(e)).

Our result showed that TGF-*β* was the most strongly elevated among three fibrogenic cytokines, and EFAX treatment ameliorated this increase more effectively than the increases in the other two fibrogenic cytokines. BAMBI and Smad7 play important roles in TGF-*β* signal transduction in the context of the pathological development of liver fibrosis. BAMBI inhibits the TGF-*β* receptor, and Smad7 acts as a TGF-*β* inhibitor, which can act as a negative feedback mechanism for TGF-*β* signaling [38]. Our data from gene expression results well reflected that the EFAX exerted upregulation of the antihepatic fibrotic genes, such as BAMBI and Smad7 (Figure 7(b)). The above results were supported by the observed gene expression levels of collagen type 1a1 and *α*-SMA, which are potent markers of the HSC activation. The gene expression levels of collagen type 1a1 and *α*-SMA were markedly upregulated in hepatic tissue by DMN injection, as has been observed in previous studies [39, 40]. EFAX treatment showed strong antihepatofibrotic effects, normalizing the altered expression levels of the above two genes (Figure 7(a)).

Regarding liver fibrosis, collagen generation and degradation are known to be very dynamic processes and are mediated by MMPs and TIMPs. ECMs are principally degraded by MMPs, whereas TIMPs are potent inhibitors of MMPs [41]. Therefore, the balance between MMPs and TIMPs is crucial in collagen degradation [42]. In the present study, we measured the protein or mRNA expression levels of MMP-2 and TIMP-1. DMN injection led to dramatic activation of TIMP-1 at both the protein and gene expression level, and MMP-2 gene expression was upregulated. The upregulation of MMP-2 gene expression may be a compensatory response to the excessive accumulation of ECM during liver fibrosis, which has been observed by other authors [43]. Treatment with EFAX is thought to activate the degradation of ECM, a hypothesis that was supported by both the suppression of TIMP-1 gene expression and the upregulation of MMP-2 gene expression (Figures 6(d) and 7(b)).

In fact, our previous studies demonstrated the antihepatofibrotic properties of* Amomum xanthioides* using a water extract (WAX) and a methanol fraction (MFAX). Both WAX and MFAX attenuated the hepatofibrotic alterations via modulation of antioxidant and anti-inflammatory effects [11, 44]. However, WAX and MFAX were potent in their antihepatofibrotic activity near a dose of 100 mg/kg, whereas a very low dose of EFAX (25 mg/kg) showed notable activity in the present study. In all of the above experiments, the EFAX 25 group generally showed notable effectiveness on parameters of hepatofibrotic activity. These data would strongly provide the clinical relevance of traditional use of* A. xanthioides*. Silymarin is a compound derived from Milk thistle and is the most well-known hepatoprotective agent [45, 46]. In our current study, silymarin treatment showed the positive effects on especially three fibrogenic cytokines but no effects on hepatic enzymes and MDA content unexpectedly. This reason is uncertain, which would be associated with the low dose (50 mg/kg) in current animal model.

Based on UHPLC-MS/MS data and* in vitro* assays, we found that EFAX contained most flavonoid contents (Figures 2(a)–2(d) and 3(a)). In contrast, the total phenolic contents of EFAX were approximately half those in MFAX (Figure 3(b)). The antioxidant capacity of WAX, MFAX, and EFAX was very similar in assays of both DPPH activity and total antioxidant capacity (data not shown); however, under TGF-*β*1 stimulation, EFAX showed the strongest activity on the gene expression levels of collagen type 1 and *α*-SMA in HSC-T6 cells, a rat derived-HSC cell line (Figures 3(c) and 3(d)). Concentration of WAX, MFAX, and EFAX treated in HSC-T6 was decided by cytotoxicity assay (Supplementary Figure 1 in the Supplementary Material available online at http://dx.doi.org/10.1155/2016/6014380).

Taken together, we conclude that the ethyl acetate fraction of* Amomum xanthioides* has potent antihepatofibrotic properties, and the underlying mechanisms involve the inactivation of HSCs via the regulation of fibrogenic cytokines, especially TGF-*β*.

## Supplementary Material

Supplementary Figure 1. Cytotoxicity of WAX, MFAX and EFAX in HSC-T6 cells. Cytotoxicity was determined in HSC-T6 cells using a WST assay. HSC-T6 Cells (2 × 10^3^) were seed in 96-well plate with DMEM. Next, each concentration of WAX, MFAX or EFAX are treated for 24 h. The data expressed as the mean ± SD (*n* = 6). ^##^ *p* < 0.01, compared with the 0 h group; ^*^ *p* < 0.05, ^**^ *p* < 0.01, compared with the non-treatment group.

## Figures and Tables

**Table 1:** Primer sequences used in this experiment.

Gene name	Primer sequence
Collagen 1a1	5′-GAT CCT GCC GAT GTC GCT AT-3′
	3′-TGT AGG CTA CGC TGT TCT TGC A-5′

*α*-SMA	5′-GAC CCT CTT CCA GCC ATC TTT-3′
	3′-GTC CTT CCT GAT GTC AAT ATC ACA CT-5′

TGF-*β*	5′-AGG AGA CGG AAT ACA GGG CTT T-3′
	3′-AGC AGG AAG GGT CGG TTC AT-5′

PDGF-BB	5′-ACC ACT CCA TCC GCT CCT TT-3′
	3′-TGT GCT CGG GTC ATG TTC AA-5′

CTGF	5′-GTG TGT GAT GAG CCC AAG GA-3′
	3′-CAG TTG GCT CGC ATC ATA GTT G-5′

TIMP-1	5′-ATG GAG AGC CTC TGT GGA TAT GTC-3′
	3′-AGG CAG TGA TGT GCA AAT TTC C-5′

MMP-2	5′-TGT GGC AGC CCA TGA GTT C-3′
	3′-TCG GAA GTT CTT GGT GTA GGT GTA-5′

Smad7	5′-TGC AAC CCC CAT CAC CTT AG-3′
	3′-GAC AGT CTG CAG TTG GTT TGA GA-5′

BAMBI	5′-TTA TGT TGG CCT TGC GAA TG-3′
	3′-TGG TGT CCA TGG AAG CTG TAG T-5′

*β*-actin	5′-AGG CCA ACC GTG AAA AGA TG-3′
	3′-CCA GAG GCA TAC AGG GAC AAC-5′

**Table 2:** Organ weights and serum biochemistries.

	Naive	DMN	EFAX 25	EFAX 50	Sily 50
Total body mass (g)	334.50 ± 18.61	243.30 ± 29.76^###^	286.00 ± 17.75	267.33 ± 31.29	268.83 ± 28.09
Absolute liver mass (g)	10.31 ± 0.88	8.82 ± 1.70	9.76 ± 1.15	9.60 ± 1.07	9.88 ± 1.68
Relative liver mass (g/100 g)	3.07 ± 0.16	3.61 ± 0.37^#^	3.41 ± 0.28	3.60 ± 0.22	3.67 ± 0.46
Absolute spleen mass (g)	0.76 ± 0.06	1.44 ± 0.27^###^	1.41 ± 0.21	1.25 ± 0.26	1.86 ± 0.35
Relative spleen mass (g/100 g)	0.23 ± 0.02	0.59 ± 0.10^###^	0.50 ± 0.08	0.48 ± 0.16	0.70 ± 0.15

AST (IU/L)	173.33 ± 24.22	848.00 ± 421.51	326.66 ± 76.33	432.00 ± 106.16	1100.00 ± 715.29
ALT (IU/L)	33.33 ± 10.32	514.00 ± 162.26^###^	226.66 ± 70.89^*∗*^	388.00 ± 140.60	513.33 ± 258.97
Total bilirubin (g/dL)	0.10 ± 0.00	1.20 ± 0.54^###^	0.36 ± 0.05^*∗∗∗*^	0.40 ± 0.08^*∗∗∗*^	1.20 ± 0.15
Platelet (k/*μ*L)	933.83 ± 41.16	222.83 ± 29.68^###^	550.66 ± 164.00^*∗∗∗*^	368.66 ± 215.3	271.50 ± 106.87

^#^ *p* < 0.05, ^###^ *p* < 0.001 compared to Naïve group; ^*∗*^ *p* < 0.05, ^*∗∗∗*^ *p* < 0.001 compared to the DMN group (*n* = 6).

## Abbreviations

*A. xanthioides*: * Amomum xanthioides*
ANOVA: One-way analysis of variance
AST: Aspartate transferase
ALT: Alanine transferase
*α*-SMA: *α*-smooth muscle actin
BAMBI: Bone morphogenic protein and activin membrane bound inhibitor
CTGF: Connective tissue growth factor
DMN: Dimethylnitrosamine
DW: Distilled water
EFAX: Ethyl acetate fraction of* Amomum xanthioides*
ECM: Extracellular matrix protein
HSC: Hepatic stellate cell
MFAX: Methanol fraction of* Amomum xanthioides*
MDA: Malondialdehyde
MMP: Matrix metalloprotease
PDGF: Platelet derived growth factor
SD: Sprague-Dawley
TGF-*β*: Transforming growth factor beta
TIMP: Tissue inhibitor of matrix metalloprotease
UHPLC-MS/MS: Ultra-high-performance liquid chromatography-tandem mass spectrometry
WAX: Water extract of* Amomum xanthioides*.
