# Supplementary material for: Ethyl Acetate Fraction of Amomum xanthioides Exerts Antihepatofibrotic Actions via the Regulation of Fibrogenic Cytokines in a Dimethylnitrosamine-Induced Rat Model
Source: Evid Based Complement Alternat Med. 2016 Aug 10;2016:6014380. doi: 10.1155/2016/6014380 (PMC4995331; doi:10.1155/2016/6014380)
Supplement: Supplementary file 1 — Supplementary Figure 1. Cytotoxicity of WAX, MFAX and EFAX in HSC-T6 cells. Cytotoxicity was determined in HSC-T6 cells using a WST assay. HSC-T6 Cells (2 × 10^3^) were seed in 96-well plate with DMEM. Next, each concentration of WAX, MFAX or EFAX are treated for 24 h. The data expressed as the mean ± SD (n = 6). ## p < 0.01, compared with the 0 h group; * p < 0.05, ** p < 0.01, compared with the non-treatment group. [file 6014380.f1.pptx]

## Slide 1
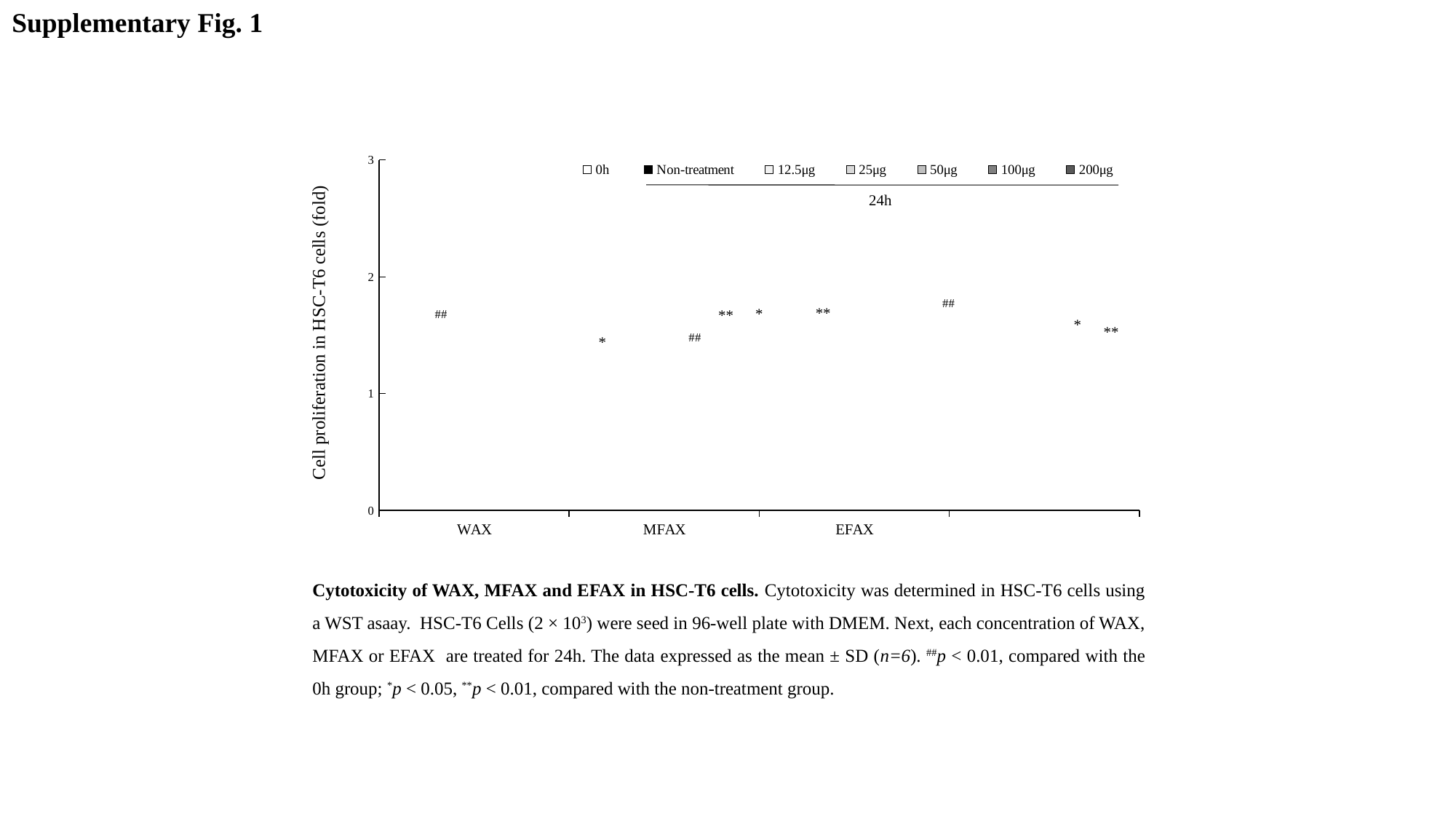

Supplementary Fig. 1
### Chart
| Category | 0h | Non-treatment | 12.5μg | 25μg | 50μg | 100μg | 200μg |
|---|---|---|---|---|---|---|---|
| WAX | 1.0 | 1.4562383612662941 | 1.4916201117318435 | 1.4432029795158285 | 1.4506517690875231 | 1.3798882681564244 | 1.3277467411545623 |
| MFAX | 1.0 | 1.3537803138373752 | 1.5492154065620543 | 1.55206847360913 | 1.5092724679029956 | 1.5506419400855922 | 1.5021398002853068 |
| EFAX | 1.0 | 1.5337331334332835 | 1.5404797601199403 | 1.5014992503748128 | 1.4508995502248878 | 1.398425787106447 | 1.325337331334333 |24h
##
**
*
**
##
*
Cell proliferation in HSC-T6 cells (fold)
**
##
*
Cytotoxicity of WAX, MFAX and EFAX in HSC-T6 cells. Cytotoxicity was determined in HSC-T6 cells using a WST asaay. HSC-T6 Cells (2 × 10^3^) were seed in 96-well plate with DMEM. Next, each concentration of WAX, MFAX or EFAX are treated for 24h. The data expressed as the mean ± SD (n=6). ##p < 0.01, compared with the 0h group; *p < 0.05, **p < 0.01, compared with the non-treatment group.
